# Dietary sodium and iodine in remote Indigenous Australian communities: will salt-reduction strategies increase risk of iodine deficiency? A cross-sectional analysis and simulation study

**DOI:** 10.1186/s12889-015-2686-1

**Published:** 2015-12-30

**Authors:** Emma McMahon, Jacqui Webster, Kerin O’Dea, Julie Brimblecombe

**Affiliations:** Wellbeing and Preventable Disease Division, Menzies School of Health Research, PO Box 41096, Casuarina, NT 0811 Australia; Centre for Population Health Research, School of Health Sciences, University of South Australia, North Tce, Adelaide, SA 5001 Australia; Food Policy Division, The George Institute for Global Health, The University of Sydney, Missenden Rd, Sydney, NSW 2050 Australia

## Abstract

**Background:**

Excess salt intake is a global issue. Effective salt-reduction strategies are needed, however, as salt is a vehicle for iodine fortification, these strategies may also reduce iodine intake. This study examines the case of the remote Indigenous Australian population; we employed an innovative, objective method to assess sodium and iodine intakes against requirements and modelled the potential effects of salt-reduction strategies on estimated sodium and iodine intakes.

**Design:**

Store-sales data were collected from 20 remote Indigenous community stores in 2012–14 representing the main source of food for 2 years for ~8300 individuals. Estimated average sodium and iodine intakes were compared against recommendations (nutrient reference values weighted to age and gender distribution). Linear programming was employed to simulate potential effects of salt-reduction strategies on estimated sodium and iodine intakes.

**Results:**

Estimated average sodium intake was 2770 (range within communities 2410–3450) mg/day, far exceeding the population-weighted upper limit (2060 mg/day). Discretionary (added) salt, bread and processed meat were the biggest contributors providing 46 % of all sodium. Estimated average iodine intake was within recommendations at 206 (186–246) μg/day. The following scenarios enabled modelling of estimated average salt intake to within recommendations: 1) 67 % reduction in sodium content of bread and discretionary salt intake, 2) 38 % reduction in sodium content of all processed foods, 3) 30 % reduction in sodium content of all processed foods and discretionary salt intake. In all scenarios, simulated average iodine intakes remained within recommendations.

**Conclusions:**

Salt intakes of the remote Indigenous Australian population are far above recommendations, likely contributing to the high prevalence of hypertension and cardiovascular mortality experienced by this population. Salt-reduction strategies could considerably reduce salt intake in this population without increasing risk of iodine deficiency at the population-level. These data add to the global evidence informing salt-reduction strategies and the evidence that these strategies can be synergistically implemented with iodine deficiency elimination programmes.

**Trial registration:**

Australian New Zealand Clinical Trials Registry: ACTRN12613000694718.

**Electronic supplementary material:**

The online version of this article (doi:10.1186/s12889-015-2686-1) contains supplementary material, which is available to authorized users.

## Background

Indigenous Australians experience premature mortality due to chronic disease at a highly disproportionate rate, and much earlier age, compared with non-Indigenous Australians [1]. Risk of cardiovascular disease (CVD) mortality in Indigenous Australians is nearly twice that of non-Indigenous Australians [2], and CVD is responsible for approximately 3 years of the life-expectancy gap experienced by this population [1]. The high prevalence of chronic kidney disease (CKD) in the Indigenous Australian population is growing concern, particularly in very remote areas; nearly four in ten Indigenous Australians living in very remote Australia have indicators of CKD [3]. Dietary improvement strategies are a priority for reducing chronic disease risk and improving health equity between Indigenous and non-Indigenous Australians.

The World Health Organisation (WHO) recommends reduction in dietary salt as a cost-effective strategy to reduce risk of chronic disease [4], and numerous studies have demonstrated the efficacy of reducing salt for lowering blood pressure [5] and other risk factors for chronic diseases such as cardiovascular disease (CVD) [6] and CKD [7, 8]. Given the high rates of hypertension, CVD and CKD in the Indigenous Australian population, particularly in remote communities [9], lowering salt intake could significantly reduce chronic disease burden.

Iodine deficiency disorders are a major public health concern, resulting in cognitive impairment, congenital abnormalities, cretinism, hypothyroidism or endemic goitre [10]. A re-emergence of iodine deficiency was observed in the early 2000’s in Australia, attributable to low usage of iodised table salt and replacement of iodine-rich sanitisers in the dairy industry [11, 12]. A study conducted in the Northern Territory in 2005–8 found that more than 40 % of Aboriginal teenagers had moderate to severe iodine deficiency (urinary iodine <50 μg/L) [13]. In 2009, mandatory iodine fortification of bread was introduced in Australia requiring iodised salt be used where salt is added to bread. Current data from the Australian Bureau of Statistics indicate that 11 % of Aboriginal and Torres Strait Islanders are iodine deficient, similar to the non-Indigenous population [9].

Monitoring population salt and iodine intakes can help to ensure that salt-reduction and iodine deficiency elimination strategies can be implemented synergistically [10]. The 2012–13 National Aboriginal and Torres Strait Islander Nutrition and Physical Activity Survey (NATSINPAS) was the first national-level dietary survey to report dietary intakes of the Indigenous Australian population [14]. However discretionary salt intake was not quantified in this survey, meaning sodium and iodine intakes are underestimated. Many remote Indigenous Australian communities have relatively closed food systems, with most of food obtained from the community store, meaning store-sales data can provide an objective indicator of community-level dietary intake over a long time period without burdening individuals in participating communities [15, 16]. We aim to examine in remote Aboriginal Australian communities using store-sales data, i) estimated adequacy (compared to population-weighted recommendations) and sources of dietary sodium and iodine, and ii) potential effects of strategies to reduce dietary sodium intake on estimated average sodium and iodine intakes. This will inform the development of tailored salt-reduction strategies for this population and identify the potential effects of these strategies on iodine adequacy.

## Methods

### Setting

Store-sales data were collected in 2012–14 from 20 stores in 20 remote communities in the Northern Territory of Australia (across Top-end and Central) where approximately 8300 individuals reside, as part of the ‘Stores Healthy Options Project in Remote Indigenous Communities’ (SHOP@RIC) [17]. This study aimed to examine the impact of a price discount intervention with or without nutrition education on purchasing of fruit, vegetables, water and diet soft-drinks in Northern Territory communities with a population of ≥100 residents and where the community store was i) managed by either Arnhem Land Progress Aboriginal Corporation (ALPA) or Outback Stores (OBS) and ii) located more than 20 kilometres from its nearest competitor. SHOP@RIC involved a baseline period, 6 month intervention period and post-intervention period. The present analysis includes all data prior to the intervention period, from July 2012 until December 2014. Ethical approval for SHOP@RIC was granted by the Menzies School of Health Research (HREC-2012-1711), Central Australian (HREC-12-13) and Deakin University (HREC-2012243) human research ethics committees. Informed consent to use community store sales data was granted by community store boards.

### Calculation of population requirements

Nutrient recommendations were derived from the 2006 Nutrient Reference Values (NRVs) [18]. Estimated energy requirement (EER) at a physical activity level of 1.6 (‘sedentary’; scale ranges from 1.2, ‘bed rest’ , to 2.2 ‘very active or heavy occupational work’ [18]), and height of 1.7 m for men and 1.6 m for women was used [19]. Age and gender distribution in the 20 communities was calculated from 2010 census data retrieved using Australian Bureau of Statistics table builder [20]. To determine the proportion of women aged 19–50 likely to be pregnant or breastfeeding at one time, 2010 birth data from very remote Northern Territory [21] was cross referenced to the total population in very remote Northern Territory in 2010 [22].

Population-weighted NRVs were calculated by multiplying the recommendation for each age/gender group by the calculated sample size in that age/gender group, summing these values and dividing by the total sample size. The midpoint for sodium adequate intake (AI) was used. NRVs for sodium and iodine (mg Na/day and μg I/day respectively) were divided by the EER (MJ/day) to get a recommended nutrient density for sodium (mg/MJ) and iodine (μg/MJ).

### Collection of apparent consumption data

Store-sales data on all food and beverage items purchased over the study period from the stores (and, where applicable, the associated takeaway food outlet) were collected by ALPA/OBS. These were imported with product codes, quantities sold and weight into a Microsoft Access database (the RIST Tool). Food items were categorised into food groups using the Australian Health Survey Food and Supplement Classification system and linked to nutrient data from the Australian Food and Nutrient survey specific database (AUSNUT 2011–13) [23].

Total sodium (mg) and iodine (μg) content of all foods and drinks purchased over the study was calculated and was divided by total energy (MJ) to give the average sodium and iodine density (mg Na/MJ and μg I/MJ respectively).

Outliers were identified by plotting weekly store-sales data for each store and matching periods of unusual data to contextual data (e.g. events in the community) to identify periods where store-sales data would not correlate with usual intake in the community (e.g. due to high influx of people who would not normally reside in the community).

### Dietary modelling

‘Estimated average intake’ was calculated by multiplying the sodium/iodine density from store-sales data by the population-weighted EER, and therefore this term refers to the amount of sodium/iodine the average person meeting the recommended EER would consume.

Linear programming was used to model the potential effects of five salt-reduction scenarios on estimated average sodium and iodine intakes. In a previous analysis, we found that it was not possible to model salt intake to below the upper limit (UL) in remote Indigenous communities through dietary changes without considerably increasing cost [24]. As most sodium comes from processed foods [25], current focus of population salt-reduction strategies is on product reformulation [26]. Therefore the present dietary modelling analysis focussed on reducing salt content of the major contributors of salt (except for discretionary salt where sodium content cannot be reduced without replacing with another mineral). The following scenarios were modelled: Scenario 1—Reduced discretionary salt intake; Scenario 2—Reduced sodium in bread; Scenario 3—Reduced discretionary salt intake combined with reduced sodium in bread (combined Scenarios 1 & 2); Scenario 4—Reduced sodium in all processed foods; Scenario 5—Reduced sodium in all processed foods and reduced discretionary salt intake (combined scenarios 1 & 4). Further information on food categorisation for dietary modelling is provided in Additional file 1. For each scenario optimisation modelling was performed to find the reduction needed to lower salt intake to within recommendations. Modelling of incremental reductions (10, 25 and 50 %) were also performed for each scenario; the results of which are shown in Additional file 1.

### Data analysis

Data were analysed using Excel 2010 (Microsoft Corporation, USA) and Stata 14 (StataCorp LP, USA). Data are presented as the total population combined with values in parentheses indicating ranges between communities.

## Results

One hundred four weeks of store-sales data for each store were available for analysis. Two outlying weeks were identified (both from the same store due to an event in the community) and excluded from the analyses.

### Apparent consumption compared to population-weighted NRVs

**Fig. 1:**
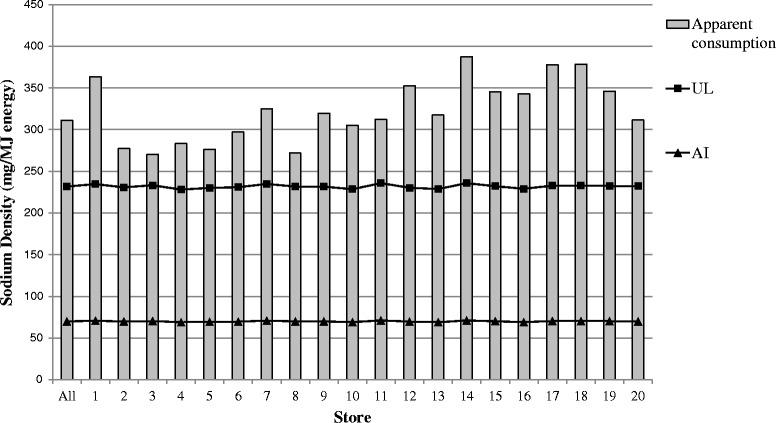
Sodium density (mg/MJ energy) of foods and drinks purchased in 20 remote Indigenous Australian communities and population weighted recommendations. Abbreviations - AI—adequate intake, MJ = megajoule, UL—Upper limit

**Fig. 2:**
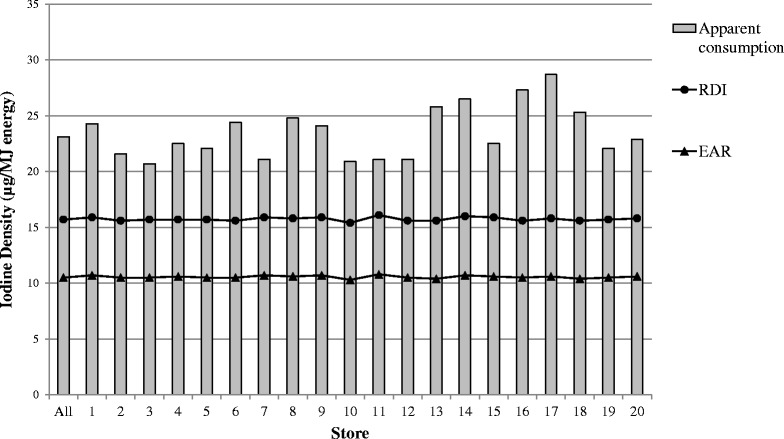
Iodine density (μg/MJ energy) of foods and drinks purchased in 20 remote Indigenous Australian communities and population weighted recommendations. Iodine density did not approach the upper limit (range from 83 to 100 μg/MJ). Abbreviations - EAR—estimated average requirement, MJ = megajoule, RDI—recommended dietary intake

Population-weighted NRVs are shown in Table 1. Average sodium densities of foods/drinks purchased varied between individual stores (Fig. 1), however all provided sodium at a density that exceeded the UL. Estimated average iodine intakes were within recommendations (Fig. 2).

**Table 1:** Apparent consumption data and population-weighted nutrient reference values for sodium and iodine in 20 remote Indigenous communities

Nutrient (unit)	Apparent consumption		Population-weighted NRV			% of recommendation
	Per MJ	Per day*	NRV	Per MJ*	Per day	
Sodium (mg)	311.0 (270.5–387.5)	2770 (2410–3450)	AI	70.0 (68.9–71.2)	620 (590–640)	444 (384–545) %
			UL	231.8 (228.2–235.9)	2060 (1950–2130)	134 (116–164) %
Iodine (μg)	23.1 (20.7–28.7)	206 (186–246)	UL	96.9 (87.4–102.6)	862 (734–927)	24 (20–31) %
			RDI	15.7 (15.4–16.1)	140 (131–145)	147 (131–182) %
			EAR	10.5 (10.3–10.8)	94 (87–98)	219 (194–271) %

Values are shown as total population (range for results from individual communities). * Per MJ of estimated energy requirement. *Abbreviations: AI* adequate intake, *EAR* estimated average requirement, *EER* estimated energy requirement, *MJ* megajoule, *NRV* nutrient reference value, *RDI* recommended dietary intake, *UL* Upper limit

### Sources of sodium and iodine

Table 2 shows sources of sodium and iodine. Discretionary (added) salt, bread and processed meat were the biggest contributors to salt intake providing 46.5 % (44.1–64.5 %) of all sodium. Major sources of iodine were bread, iodised table salt, milk and eggs, providing 80.7 % (72.7–89.4 %) of iodine. Iodised table salt was the most commonly purchased salt representing 71.7 % (38.2–96.6 %) by weight of all salt purchased.

**Table 2:** Sources of sodium and iodine

Category	Na % (range)	Na (mg/100 g)	I % (range)	I (μg/100 g)
**Herbs, spices, seasonings & stock cubes**	**19.3 (12.4–38.6)**	**32,544**	**21.6 (14.2–39.8)**	**2693**
Salt	18.9 (12.1–38.4)	37,303	21.6 (14.2–39.8)	3162
*Salt, table, iodised*	*13.9 (9.0–26.9)*	*38,168*	*21.5 (14.1–39.8)*	*4400*
*Salt, table, non-iodised*	*3.3 (0.0–11.5)*	*38,178*	*0.0 (0.0–0.1)*	*20*
**Regular breads & bread rolls**	**18.3 (13.3–25.0)**	**445**	**34.4 (23.6–44.5)**	**62**
White breads	0.9 (0.1–2.3)	459	1.4 (0.2–3.5)	56
Wholemeal breads	15.5 (10.4–21.1)	443	29.1 (17.9–37.6)	62
Mixed grain breads	1.9 (0.2–6.2)	459	3.9 (0.3–15.3)	70
**Processed meats**	**8.9 (5.9–15.0)**	**779**	**0.8 (0.5–1.7)**	**6**
Canned meat	5.5 (2.6–11.3)	643	0.7 (0.4–1.5)	6
Bacon	1.2 (0.6–2.9)	1289	0.0 (0.0–0.0)	1
Ham	1.0 (0.3–3.8)	1257	0.1 (0.0–0.3)	8
**Dried & preserved fruit**	**4.5 (1.5–9.3)**	**5642**	**0.0 (0.0–0.1)**	**4**
Plum, salted	4.5 (1.5–9.3)	8400	0.0 (0.0–0.1)	4
**Mixed dishes where cereal is the major ingredient**	**4.1 (2.6–8.5)**	**451**	**1.9 (0.6–5.1)**	**16**
Savoury pasta/noodle & sauce dishes	1.2 (0.1–4.4)	582	1.2 (0.1–4.0)	42
Sandwiches & filled rolls	1.4 (0.9–2.8)	342	0.1 (0.1–0.3)	3
**Pastries**	**4.0 (2.4–8.9)**	**500**	**0.7 (0.3–1.7)**	**6**
Savoury pastry products, pies, rolls & envelopes	3.5 (2.3–8.1)	490	0.6 (0.3–1.5)	6
**Gravies & savoury sauces**	**3.5 (2.3–8.5)**	**1611**	**0.1 (0.0–0.2)**	**4**
Savoury sauces, not tomato based, commercial	1.6 (0.5–6.2)	3159	0.0 (0.0–0.1)	5
Savoury sauces, tomato based, commercial	1.0 (0.5–2.0)	756	0.1 (0.0–0.1)	4
**Dairy milk (cow, sheep & goat)**	**3.4 (2.3–7.3)**	**170**	**18.6 (12.3–30.8)**	**70**
Milk, powder, cow, dry	3.0 (1.9–7.0)	311	15.6 (10.1–28.8)	121
Milk, cow, fluid	0.4 (0.2–0.9)	37	2.9 (1.3–8.1)	22
**Pasta and pasta products (without sauce)**	**3.0 (1.7–4.6)**	**619**	**0.1 (0.1–0.2)**	**2**
Instant noodles and noodle products, wheat based	3.0 (1.7–4.6)	676	0.1 (0.0–0.1)	1
**Soft drinks, & flavoured mineral waters**	**2.2 (1.6–3.5)**	**15**	**3.4 (2.5–5.1)**	**2**
**Eggs**	**1.2 (0.9–1.9)**	**150**	**6.0 (4.0–9.0)**	**57**

Percentages of intake are of the total dietary intake of that nutrient. Ranges are for values from individual communities. Not all food types are shown; the food or drink categories shown in this table represent 72.5 % (range between individual communities 72.3–86.3 %) of total sodium and 87.8 (83.4–95.5)% of total iodine.Sub-major categories (bolded) that provide ≥3 %, and minor categories (not bolded) that provide ≥1 % of sodium or iodine from the AUSNUT classification system [23] are shown. Foods within 'salt' minor food group [23] that provide ≥1 % of sodium or iodine are also shown (italicised). Some categories have been combined and category names have been modified for brevity.

### Dietary modelling

Neither Scenario 1 (reduction in discretionary salt intake) nor Scenario 2 (reduction in sodium content of bread) were sufficient to reduce estimated average sodium intake to below the population-weighted UL, even if 100 % reduction was applied (Scenario 1: 2250 mg Na, 161 μg I; Scenario 2: 2230 mg Na and 133 μg I/day). However when these strategies were combined (Scenario 3), a 67 % reduction in both sodium content of bread and discretionary salt intake could reduce sodium intake to below the UL (estimated average intake 2060 mg Na and 127 μg I/day).

Wide-spread changes across processed foods were modelled in Scenario 4 (reduced salt content of all processed foods) and Scenario 5 (reduced salt content of all processed foods plus reduced discretionary salt intake). For estimated average sodium intake to be below the population-weighted UL, 38 % reduction needed to be applied in Scenario 4 (2060 mg Na and 175 μg I/day), and a 30 % in Scenario 5 (2050 mg Na and 168 μg I/day).

## Discussion

We collected 2 years of store-sales data from 20 remote Indigenous community stores representing the main source of food for approximately 8300 individuals as an objective indicator of usual dietary intake. This is the largest apparent consumption dataset for dietary intake specific to those living in remote Indigenous communities to date, and the first study in this population examining the potential effects of salt-reduction strategies on sodium and iodine intakes. We found that estimated average sodium intake was above that recommended while iodine intake was within recommendations. The 2012–2013 NATSINPAS measured self-reported dietary intake of 4100 Indigenous Australians including 2300 living in remote Australia (although did not differentiate between remote and very remote) using 24-h recall. Self-reported dietary sodium and iodine intakes were 2096 mg and 145 μg/day respectively for those living in remote Australia, however these estimates do not include sodium and iodine from discretionary salt, which contributed 19 % of sodium and 21 % of iodine in the present study. Excluding sodium and iodine from discretionary salt, our estimated average intakes (2263 mg Na and 162 μg/I) are within 5 % of the estimates from the NATSINPAS (2378 mg Na and 164 μg I [14]).

We found that the biggest contributors to salt intake were discretionary salt (20 %), bread (18 %) and processed meat (9 %; ~6 % of which was canned meat). This is consistent with findings from the NATSINPAS; bread and processed meat contributed 20 and 11 % of all sodium for Indigenous Australians in remote setting, (or 16 and 9 % if adjusting for estimated discretionary salt intake) [14]. Major sources of iodine were bread (35 %), iodised table salt (21 %), milk (19 %) and eggs (6 %). In the NATSINPAS, bread contributed 34 % of iodine, milk 14 % and eggs 5 % (adjusted values 28, 11 and 4 % respectively) for those living remotely.

This analysis highlighted the need for salt-reduction strategies in remote Indigenous communities to reduce salt intakes to within recommended levels. High sodium intakes are prevalent in most populations with access to processed foods [27], and a similar sodium intake was found when we previously characterised nutrient intake in a smaller number of remote Indigenous communities [24], therefore the excess sodium intake found in this setting is not surprising. We previously modelled dietary changes to optimise diet quality in remote Indigenous communities, and found that even with large shifts from processed to unprocessed foods, sodium intake remained above the upper limit [24]. Sodium is ubiquitous throughout the food supply and achieving sustained behaviour change to reduce salt intake is challenging [27]. As most sodium comes from processed foods, focus has turned to food reformulation as a solution to reduce population salt intake [27]. In 2009, the Australian Food and Health Dialogue was established and voluntary food reformulation targets were set for maximum sodium levels across a range of commonly consumed foods [28]. However sodium contents of many products are still above these targets [26].

Bread is consistently found to be one of the biggest contributors to sodium intake in Australia and other westernised countries, attributable to the moderate-high sodium content of bread and regular consumption [27]. Sodium content of bread in the present study ranged from 330 to 790 mg Na/100 g for individual products, whereas the target for the maximum salt content of bread set by the Australian Food and Health Dialogue is 400 mg/100 g [28], suggesting opportunity for further salt-reduction. Modelling reduction in salt content of bread resulted in small reductions in estimated salt intake, similar to those when reduced discretionary salt intake alone was modelled, however either strategy individually could not reduce sodium intake to below the UL. Combining these two strategies could reduce average sodium intake to below the UL, however large (67 %) reductions were needed.

Wide-spread reduction across all processed foods could also reduce estimated salt intakes to within recommendations. Approximately 40 % reduction was needed before the estimated average sodium intake was below the UL. If reduction occurred concurrently with reduced discretionary salt intake, 30 % reduction in both sodium content of processed foods and discretionary salt intake would reduce estimated average sodium intake to below the UL. Such reductions are not unprecedented [27, 29]. The United Kingdom has achieved reductions in key food products of between 25 and 45 %. This along with a social marketing campaign and labelling changes to target behaviour change to reduce salt intake achieved a 15 % reduction from 9.5 to 8.1 g/day over 10 years [30]. This was mirrored by blood pressure reduction of 3/1 mm Hg after adjusting for known confounding factors (including fruit and vegetable intake, body mass index and alcohol intake) and a 36 % reduction of both stroke and ischaemic heart disease mortality [30].

Implementing practical and effective strategies to reduce salt intake is imperative; CVD is the leading cause of mortality in Indigenous Australians [2], and high blood pressure is one of the strongest predictors of cardiovascular events [31]. He and Macgregor estimated that a 6 g/day reduction in salt intake (equivalent to 2300 mg sodium) in the UK could reduce systolic/diastolic blood pressure by 5/3 mm Hg reducing risk of stroke by approximately 25 % and ischemic heart disease (IHD) by 18 %, while a 3 g/day (1150 mg sodium) reduction could reduce blood pressure by 2/1 reducing stroke by 13 % and IHD by 10 % [32]. A reduction in dietary salt as small as 0.5 g/day (200 mg sodium) could have significant outcomes in terms of reduced CVD mortality at the population level [32]. Since sodium balance is a main role of the kidney, those with kidney impairment may be particularly ‘salt-sensitive’ and therefore more susceptible to the adverse effects of excess salt intake [7]. Nearly 40 % of the remote Indigenous Australian population have indicators of CKD [9], therefore population salt-reduction may be particularly beneficial in reducing risk of CVD and CKD progression.

Iodine fortification was an important contributor to iodine intakes in this population as evidenced by half of all dietary iodine coming from the main two targets for iodine fortification (bread and iodised salt). Despite this, iodine was not reduced to below the EAR in any of the modelled scenarios of salt-reduction. This is consistent with previous research showing that salt reduction does not increase iodine deficiency [10], or that iodine status does not differ across levels of salt intake [33]. It is important that the messages for salt-reduction and iodine deficiency prevention are consistent [34]. The WHO recommends this be achieved by: 1) clear non-conflicting messages around iodised salt usage (an example used in Italian public health campaigns is ‘Poco sale, ma iodate!’ which translates to ‘Little salt, but all iodised’), 2) universal salt iodisation (all salt used in processed foods is iodised), and 3) increasing the iodine to sodium ratio in iodised salt as salt intakes are reduced [10]. Further, the Australian National Health and Medical Research Council (NHMRC) recommended that pregnant and lactating women take daily iodine supplementation [35].

There are several limitations to the present analysis. Although iodine intake was within recommendations at the population level, we did not measure individual level intake or the proportion of individuals at risk of deficiency; therefore inferences about iodine-sufficiency are applicable at the population-level only. The data composition tables used for this analysis provide an average of like products whereas sodium content may differ by product [23]. Store-sales data does not account for food wastage (once it has been purchased) or foods obtained from sources other than the community store (or its associated takeaway outlet) such as wild-harvested foods or municipal water. However, it is estimated that this represents only a small proportion of dietary intake [14]. Wild-harvested foods provided less than 2 % of energy and less than 1 % of sodium and iodine in the NATSNPAS remote Indigenous Australian sample, while water (municipal and bottled combined) provided 4.5 % of iodine and 1.3 % of sodium [14]. Communities included in the present study did not have food vendors in the community outside of the primary store (except for one community which had a second, smaller store open during the study). The median distance to the nearest competitor was 81.5 km (range 25–443 km).

A further limitation is that the estimated average intakes in this paper were based on an assumption that the average person was consuming sufficient energy to meet the EER, which may not be the case in a population where food insecurity is common [14]. Average reported energy intake from the NATSINPAS for Indigenous Australians living remotely was 8.5 MJ/person/day, which is 96 % of the population-weighted EER calculated for this study [14]. Further, the studied population would need to be consuming considerably under the EER for average sodium intake to be within recommendations, and 46 % (36–51 %) of the EER for the average iodine intake to be below the population-weighted EAR. A further limitation of this analysis is that ALPA and OBS have Nutrition Policies regarding availability of iodised versus non-iodised salt (whereby iodised table salt must be stocked in stores where table salt is available), which may limit transferability of the results to other communities.

## Conclusion

Modelling of multi-faceted strategies including salt-reduction of bread and other processed foods combined with reduced discretionary salt usage can reduce salt intake to within recommendations—a level likely to reduce risk of chronic disease in this population. Salt-reduction strategies can be implemented in this population without increasing risk of iodine deficiency at the population-level. This study provides an innovative, objective method for assessing sodium and iodine intakes against population-weighted requirements and for modelling the potential effects of salt-reduction strategies on dietary sodium and iodine. This adds to the global evidence informing salt-reduction strategies and the potential effects of salt reduction on iodine intakes. Salt-reduction strategies and iodine deficiency elimination programmes are both important for maintaining optimal population health and therefore it is imperative that they are implemented synergistically.

## Additional file

Additional file 1: Table S1. Scenarios modelled to reduce salt intake. **Table S2.** Dietary modelling results. (DOCX 25 kb)
