# Supplementary material for: Dietary sodium and iodine in remote Indigenous Australian communities: will salt-reduction strategies increase risk of iodine deficiency? A cross-sectional analysis and simulation study
Source: BMC Public Health. 2015 Dec 30;15:1318. doi: 10.1186/s12889-015-2686-1 (PMC4696303; doi:10.1186/s12889-015-2686-1)
Supplement: Additional file 1: Table S1. — Scenarios modelled to reduce salt intake. Table S2. Dietary modelling results. (DOCX 25 kb) [file 12889_2015_2686_MOESM1_ESM.docx]

# Additional file 1

## Table S1 - Scenarios modelled to reduce salt intake

| **Scenario** | **Categorisation** | **Types of foods** | **Salt reduction** | **Iodine reduction** |
| --- | --- | --- | --- | --- |
| **1. Reduced**  **discretionary salt intake** | **Salt** | All foods in AUSNUT ‘Salt’ group | Yes | Yes |
|  | **Not salt** | All others | No | No |
| **2. Reduced sodium in bread** | **Bread foods** | Foods where all sodium in product comes from bread dough, including regular breads/rolls, English muffins, flat breads & savoury/sweet breads. | Yes | Yes |
|  | **Bread-containing foods** | Foods where sodium comes from bread dough & other ingredients, including sandwiches, burgers, pizzas, filled wraps & breads with high-sodium toppings | Partial† | Mixed* |
|  | **Non-bread foods** | All other foods, including cakes pancakes, doughnuts, crumbed meat products, taco/burrito kits | No | No |
| **3. Combined 1 & 2** | *As per scenario 1 & 2* | | | |
| **4. Reduced sodium in processed foods** | **In** | All processed food >120mg Na/100g, where the sodium/salt is added during processing | Yes | Mixed* |
|  | **Out** | Foods <120mg Na/100g (as already low salt foods) or sodium not added during processing (including some egg products, milk powders, gelatine and some dried herbs/spices), and foods where sodium is entirely functional (includes leavening agents or sports drinks). | No | No |
| **5. Combined 1 & 4** | *As per scenario 1 & 4* | | | |

†As we could not determine the proportion of salt coming from the dough versus the other ingredients in these products, it was modelled as if half of the salt came from the bread dough, and limits of uncertainty were calculated. *Foods were classified as 1) iodine reduced in proportion with sodium reduction (foods where the iodine mostly came from iodised salt, e.g., bread products), 2) iodine was reduced at half the rate of sodium (foods where it could not be determined the extent to which iodine would be reduced with salt reduction; limits of uncertainty were calculated for analyses including these foods), or 3) iodine was not reduced with sodium reduction – foods where iodine in the product was not associated with sodium (e.g., cheese or other dairy products, seafood, seaweed containing products).

## Table S2 Dietary modelling results

| **Scenario** | **Description** | **% reduction** | **Na (mg/day)** | **I (µg/day)** |
| --- | --- | --- | --- | --- |
| **Original** | Estimated average intakes | 0% | 2770 | 205 |
| **Scenario 1** | Modelled reduced  discretionary salt intake | 10% | 2720 | 201 |
|  |  | 25% | 2640 | 194 |
|  |  | 50% | 2510 | 183 |
| **Scenario 2** | Modelled reduced sodium content of bread | 10% | 2710* | 198* |
|  |  | 25% | 2630* | 187* |
|  |  | 50% | 2500* | 169* |
| **Scenario 3** | Combined scenario 1 & 2 | 10% | 2660* | 194* |
|  |  | 25% | 2500* | 176* |
|  |  | 50% | 2240* | 147* |
| **Scenario 4** | Modelled reduced sodium content of all processed foods | 10% | 2580 | 197† |
|  |  | 25% | 2300 | 186† |
|  |  | 50% | 1840 | 166† |
| **Scenario 5** | Combined scenario 1 & 4 | 10% | 2530 | 193† |
|  |  | 25% | 2170 | 175† |
|  |  | 50% | 1580 | 144† |

* limits of uncertainty are +/- <1% to account for mixed foods. † limits of uncertainty are +/- 2.7% to account for mixed foods.
